# Let’s *not* talk about sex: Unexpected tensions in teaching women’s health

**DOI:** 10.1007/s40037-018-0423-z

**Published:** 2018-04-24

**Authors:** Helen J Reid, Jennifer L Johnston

**Affiliations:** Centre for Medical Education, Queen’s University, Belfast, UK

**Keywords:** Women’s health, Feedback, Primary care, Reflection

## The story

As clinical educators, our vocation for medicine is deeply enmeshed with our research and educational practice. In general, we have found this to add credibility and depth to our teaching of medical students. Here, though, we describe our surprise at encountering tension and pushback upon introducing something important from our clinical world to medical education. We reflect on how this situation evolved, how we resolved it, and what we have learned from it, in the hope of informing others who may find themselves in similar positions.

Working as experienced female general practitioners (GPs) in the United Kingdom, we have long been aware of challenges around diagnosis and management of women’s health conditions. This area of medicine reflects a cultural normalization of pejorative attitudes towards ‘women’s problems’, leading to well-documented health inequalities [1]. Historically, this has been perpetuated by patriarchal attitudes within society, and subsequently within gynaecology [2–4]. Even within our modern society, certain aspects of female sexual and reproductive health remain open to stigma and even ridicule [5, 6]. Clinically, this translates to ‘hard’ outcomes in terms of delayed diagnoses and significant morbidity [7, 8]. We have observed this in action in our clinical practice, and have explored it within our academic scholarship [9, 10]. As researchers, we are critical scholars in that we seek to question taken for granted assumptions, and are committed to following a social justice agenda. Additionally, this form of advocacy for patients is a central tenet of family medicine [11], and therefore finds a natural home within general practice curricula.

We wanted to incorporate these insights into our undergraduate medical program, specifically by introducing the concept of feminism in approaching women’s health in primary care. Our specific context is within a UK institution, where the GP teaching program takes place predominantly in year four of a five-year medical curriculum. Traditionally, women’s health teaching has only been directly taught during specialist obstetrics and gynaecology modules in a secondary care setting. This offers students a primarily disease-focused model. We developed and introduced a session, billed ‘Women’s health in primary care’, within the GP teaching program. In this 90-minute session, which we facilitated, we introduced a patient-focused, primary care oriented approach encompassing common gynaecological disorders, mental health issues and public health interventions. In framing the session, we explored common inequalities and stigma associated with the healthcare of women. In doing so, we made explicit and subsequently challenged traditional patriarchal discourses relative to menstruation, sex, bleeding, childbirth and menopause. We encouraged students to think around why women’s health has been repressed historically. We then explored conditions including polycystic ovarian syndrome, endometriosis, and pelvic inflammatory disease using patient narratives as exemplars and stimuli for small group work.

## Surprising outcomes

Over the course of one academic year, we delivered this session 12 times to approximately 270 students. Our intention was to spark interest and even debate amongst the students. Our first major surprise was the valency of reaction to the teaching. This was strongly polarized. Good feedback, both formal and informal (including on social media) tended towards excellent. Students who engaged with the materials and concepts reported positive learning experiences which helped them to ‘think outside the box’. On the other hand, during several of the sessions we experienced far more resistance from small subgroups of students than we are typically used to, as experienced teachers on a popular and well-received course. This resistance typically manifested as non-engagement with tasks and group discussions, sometimes associated with negative body language such as folded arms and eyes raised to the ceiling. While we made efforts to engage all members of the group, we did not compel students to participate.

Our fourth year students are usually only too willing to engage with our teaching. Some, however, appeared puzzled, even lost for words in the face of these new challenges. During lectures, these students often seemed to have difficulty postulating why and how women’s health might be affected by sociocultural or historical influences. At times, they simply appeared to us to be bored and detached, regardless of how engaged their peers might be. Getting them to consider roots of the word ‘hysterectomy’ (meaning hysteria) did not serve as the spark that we had hoped it would to provoke discussion. Explaining ‘how to be a feminist doctor’, in the broadest terms of patient-centredness and advocating for all underserved groups, evoked strong responses, bordering on incredulity. Some students reported feeling uncomfortable in the face of open discussion around sex, sexual health and behaviour, and contraceptive options. We had not anticipated so much discomfiture around frank discussion of sex.

## Lessons learned

As experienced clinicians, our first duty is to our patients, and our role is to best serve patients’ needs and address health inequalities. It is therefore, in our view, essential that we help doctors-in-training to understand structural influences on health and illness, yet we received feedback that some students found ‘politics’ in medicine uncomfortable. Having undertaken significant soul-searching, including lengthy discussion with our tolerant academic peers, we remain confident that it is our duty to introduce students to primary care medicine, and primary care medicine is inextricably political [12]. We were, perhaps, somewhat naïve in our expectation that these tenets would be universally embraced.

Having extensively explored the rationale for introducing the teaching in the first place, and found it sound and secure within a demonstrable evidence base, the next challenge was to examine the manner of its delivery. Familiar with a more didactic teaching approach, often with a heavy focus on scientific content (in keeping with Foucault’s ‘clinical gaze’ [13]) not all medical students may find the shift to primary care comfortable. We think that we underestimated how challenging it might be for students steeped in hospital medicine to encounter the different epistemology of primary care [14], and its emphasis on relational, dialogic and person-centred medical practice [15]. That prejudice and stigma should be reified in health outcomes is not surprising to established GPs, but is potentially overwhelming for a junior trainee more attuned to the relative certainty of hospital practice [16]. Indeed, it was our (anecdotal) observation that, as the year went on and subsequent cohorts of students had accrued more diverse clinical experience, we encountered far greater openness to discussion of such concepts. We recognize that for many students any deviation (real or perceived) from teaching aligned with more familiar biomedical content presents a potential challenge. Indeed, perhaps we challenged not just their knowledge base, but more profoundly their identity as fledgling clinicians.

Moving from this deconstruction of the problem towards possible solutions, as behoves our GP roots, we decided that engaging resistant students in dialogue was a good place to start. This year, we have introduced a multi-pronged approach, in an attempt to bring students with us as we follow certain lines of logic. We now introduce the session with the disclaimer that we realize frank discussion of sexual and gynaecological health may cause students to consider their own moral position on such issues. We differentiate between our personal moral status, and our professional obligation to engage with patients without judgement. In this line, we refer students to General Medical Council (GMC) guidance on conscientious objection [17], and actively encourage questions and commentary as we go along. We have slightly softened the language we use around feminism and inequality, without diluting the content, and offer more examples from our clinical practice. We find that students are highly responsive to our own clinical experiences and love to hear these real-life narratives.

Beyond this single lecture, we have expanded on other aspects of general practice teaching, to introduce philosophy of care and epistemology earlier. This allows students to ‘follow the breadcrumbs’ throughout the course of the GP program. Advocating for person-centred care as a central tenet of the primary care paradigm runs as a theme through several teaching sessions, involving different faculty, which means that students are no longer ‘coming cold’ to a session which is radically different from the majority of their previous teaching.

Our surprise at encountering resistance led us to reflect on why and how we had introduced potentially challenging concepts. Our own reactions as educators to polarized feedback were another surprise—a minority of negative comments outweighed the positive, and led to that sinking feeling of failure. We realized that the responses to our session had taken on a personal significance for us both, cutting as it did across our multiple roles as educators, doctors, patients, and of course simply women.

## Moral of the story

Reflexivity as clinical teachers is, as with clinical practice, vitally important to optimize our work as educators. Ultimately, though, perhaps the most important lesson is that change is inherently difficult. Radical challenge to the status quo will perhaps inevitably be met with resistance; not everyone will ‘come with you’. Yet like many of our fellow educators, we have tended to pay greater attention to our negative feedback than the largely very positive response which we also encountered. Do we simply need to learn that, after all due reflection and adjustments have been made, sometimes it is about sticking to our well-argued, evidence-based guns?

In today’s healthcare world, helping students to a critical appreciation of their role as practitioners can only be of benefit to their patients. We share this anecdote then, with a somewhat wry smile, in the hope that at least some of our colleagues will recognize this experience and take heart that, in the long run, it is all worth it.
